# Basal Cell Carcinoma with Myoepithelial Differentiation: Case Report and Literature Review

**DOI:** 10.7759/cureus.2081

**Published:** 2018-01-17

**Authors:** Philip R Cohen

**Affiliations:** 1 Department of Dermatology, University of California, San Diego

**Keywords:** actin, basal, carcinoma, cell, differentiation, myoepithelial, muscle, smooth

## Abstract

Basal cell carcinoma is the most common skin cancer. Myoepithelial cells are specialized epithelial cells. Basal cell carcinoma with myoepithelial differentiation is a rare tumor. A 71-year-old man with a basal cell carcinoma with myoepithelial differentiation that presented as an asymptomatic red papule of two months duration on his forehead is described. Including the reported patient, this variant of basal cell carcinoma has been described in 16 patients: 11 men and five women. The patients ranged in age at diagnosis from 43 years to 83 years; the median age at diagnosis was 66 years. All of the tumors were located on the face—most were papules or nodules of less than 10 x 10 mm. Their pathology demonstrated two components: one was that of a typical basal cell carcinoma and the other was myoepithelioma-like in which the tumor cells were plasmacytoid or signet ring in appearance and contained abundant eosinophilic cytoplasm or hyaline inclusions or both. The myoepithelial tumor cells had variable immunohistochemical expression that included not only cytokeratin but also actin, glial fibrillary acid protein, S100, and vimentin. The most common clinical impression, prior to biopsy, was a basal cell carcinoma. The pathologic differential diagnosis included cutaneous mixed sweat gland tumor of the skin, myoepithelioma, myoepithelial carcinoma, and tumors that contain a prominent signet ring cell component (such as metastatic gastrointestinal and breast carcinoma, melanoma, plasmacytoid squamous cell carcinoma, and T-cell lymphoma). Mohs micrographic surgical excision, with complete removal of the tumor, was recommended for treatment of the carcinoma.

## Introduction

Basal cell carcinoma with myoepithelial differentiation is a rare pathologic variant of this cutaneous neoplasm. The features of a 71-year-old man with basal cell carcinoma with myoepithelial differentiation presenting as a nodular tumor on his forehead are described. The clinical and pathology characteristics of patients with basal cell carcinoma with myoepithelial differentiation are reviewed [1-10].

## Case presentation

A 71-year-old man presented for evaluation of an asymptomatic red lesion of two months duration on his forehead. He previously had actinic keratoses on his face that were treated with cryotherapy; however, he had never had a non-melanoma skin cancer. His medical history was significant for a bradycardia (secondary to a first-degree atrioventricular block), hypothyroidism, mixed hyperlipidemia, and prediabetes (controlled by diet); his medications included atorvastatin and levothyroxine.

**Figure 1:**
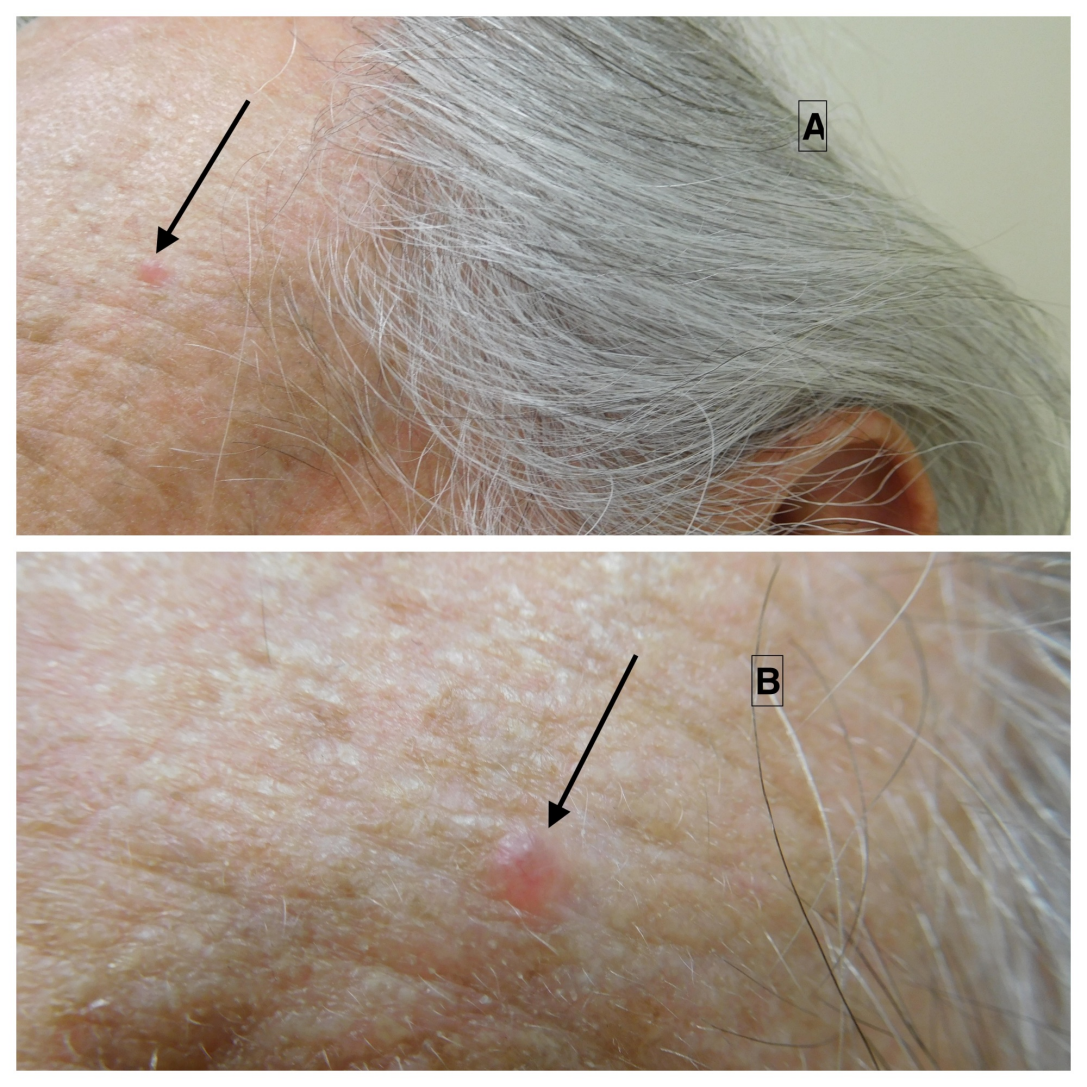
Clinical features of basal cell carcinoma with myoepithelial differentiation. Basal cell carcinoma with myoepithelial differentiation presenting as an asymptomatic 3 x 3 mm papule on the left forehead of a 71-year-old man.

Cutaneous examination of his face showed a 3 x 3 mm erythematous papule on the left side of his forehead (Figure 1). The clinical differential diagnosis included basal cell carcinoma, an adnexal cyst, and an inflammatory papule. A shave biopsy was performed.

**Figure 2:**
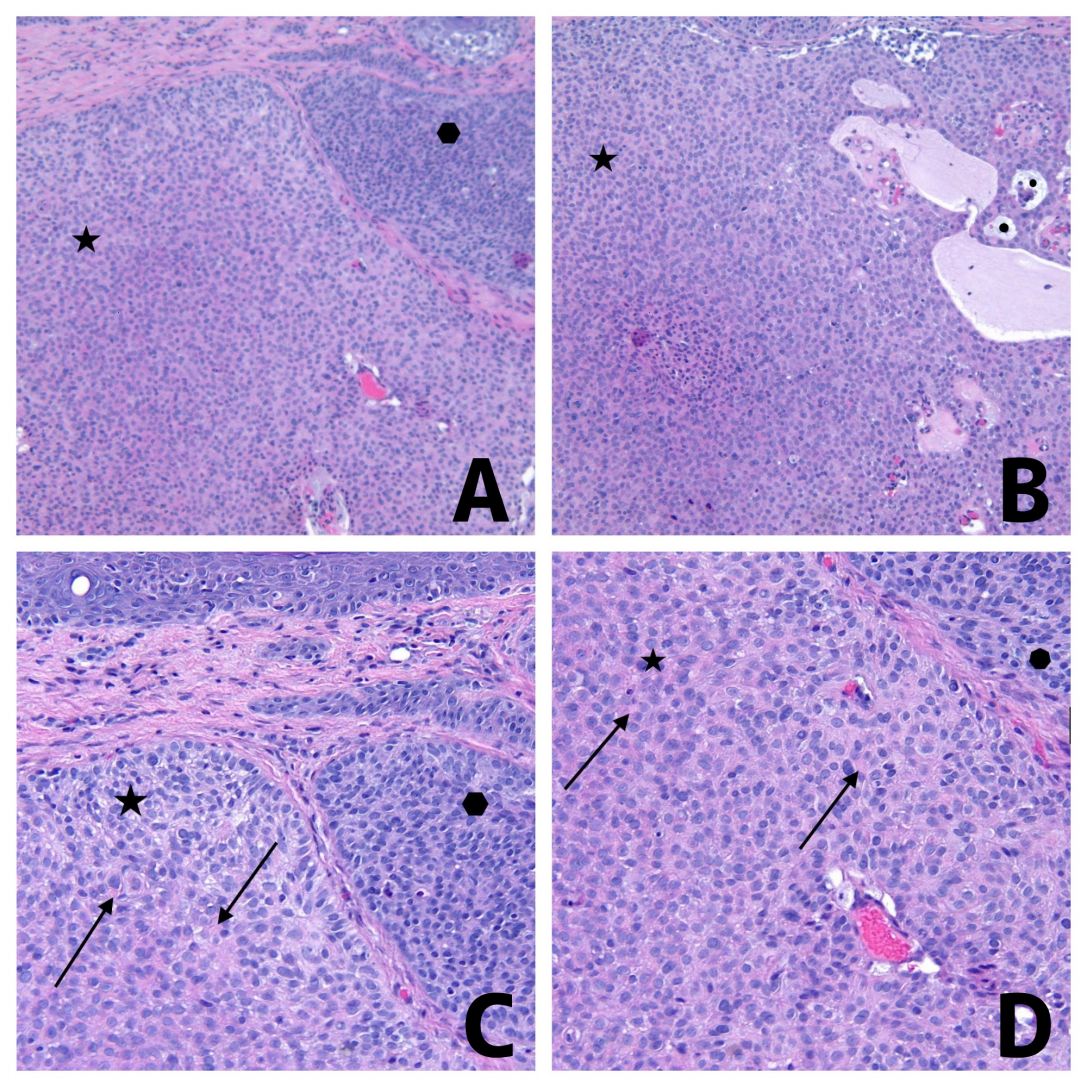
Pathology features of basal cell carcinoma with myoepithelial differentiation. Distant (A and B) and closer (C and D) view of hematoxylin and eosin stained sections of a basal cell carcinoma with myoepithelial differentiation from the left forehead of a 71-year-old man. The dermal tumor consists of multinodular aggregates (A); some of the aggregates of tumor cells have basophilic cytoplasm (solid hexagon) whereas other aggregates of tumor cells contained eosinophilic cytoplasm with hyaline globules (solid star). The aggregates of tumor cells surround collections of mucin (solid circles) (B). Hyaline globules are present in the eosinophilic cytoplasm of the tumor cells with myoepithelial differentiation (solid arrows) (C and D) [Hematoxylin and eosin: A, x2; B, x2; C, x10; D, x20].

Microscopic examination of hematoxylin and eosin stained sections of the lesion showed a tumor extending from the epidermis into the dermis and consisting of atypical basaloid cells, with peripheral palisading of the nuclei, in multinodular aggregation (Figure 2). Some of the aggregates of tumor cells had basophilic cytoplasm. However, other aggregates of tumor cells contained eosinophilic cytoplasm or hyaline globules or both. The tumor cells surrounded collections of mucin.

**Figure 3:**
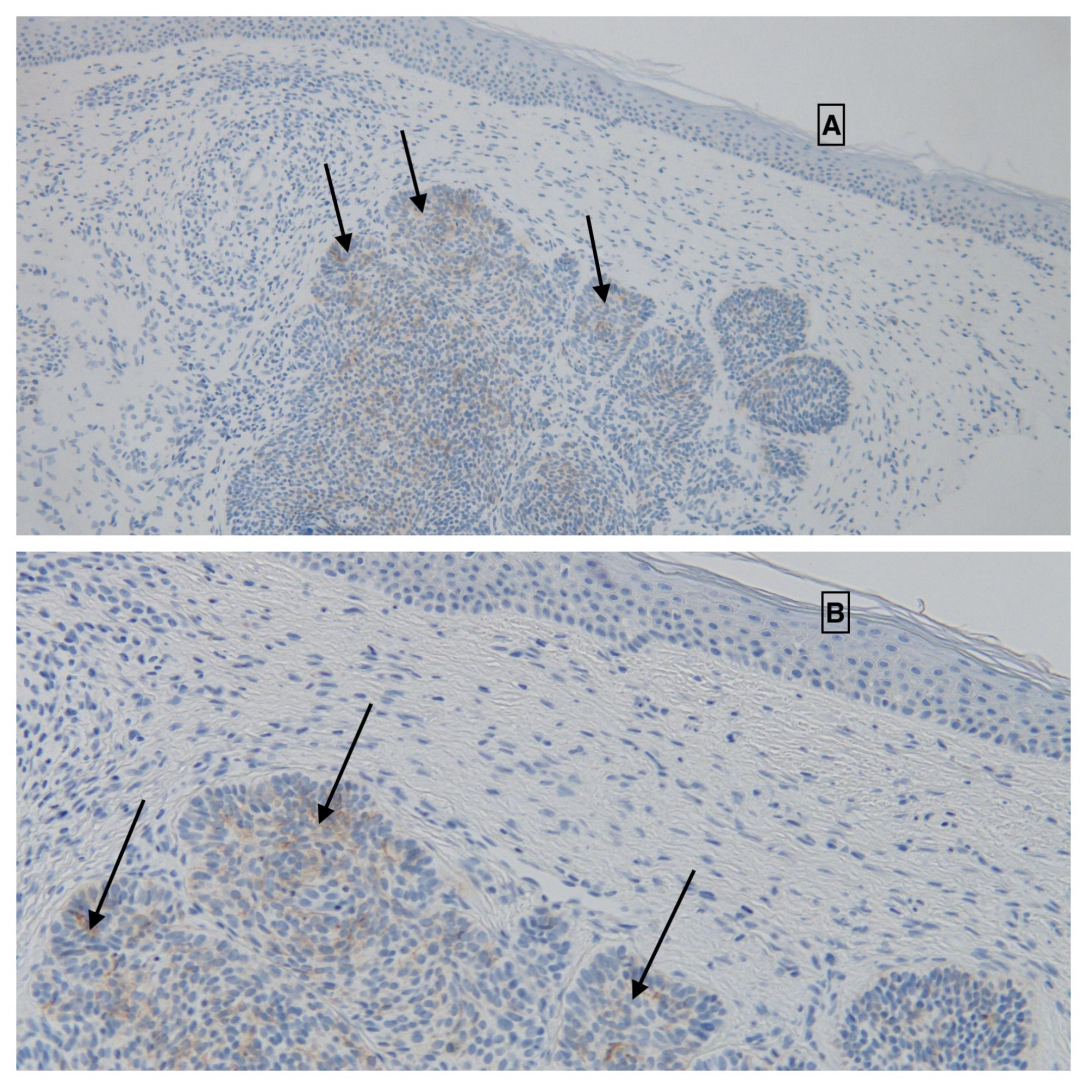
Immunoperoxidase studies of basal cell carcinoma with myoepithelial differentiation: Ber-EP4. Distant (A) and closer (B) view of Ber-EP4 expression (solid arrows) of a basal cell carcinoma with myoepithelial differentiation. The positive staining confirms the diagnosis of basal cell carcinoma [Immunoperoxidase, Ber-EP4: A, x2; B, x 10].

**Figure 4:**
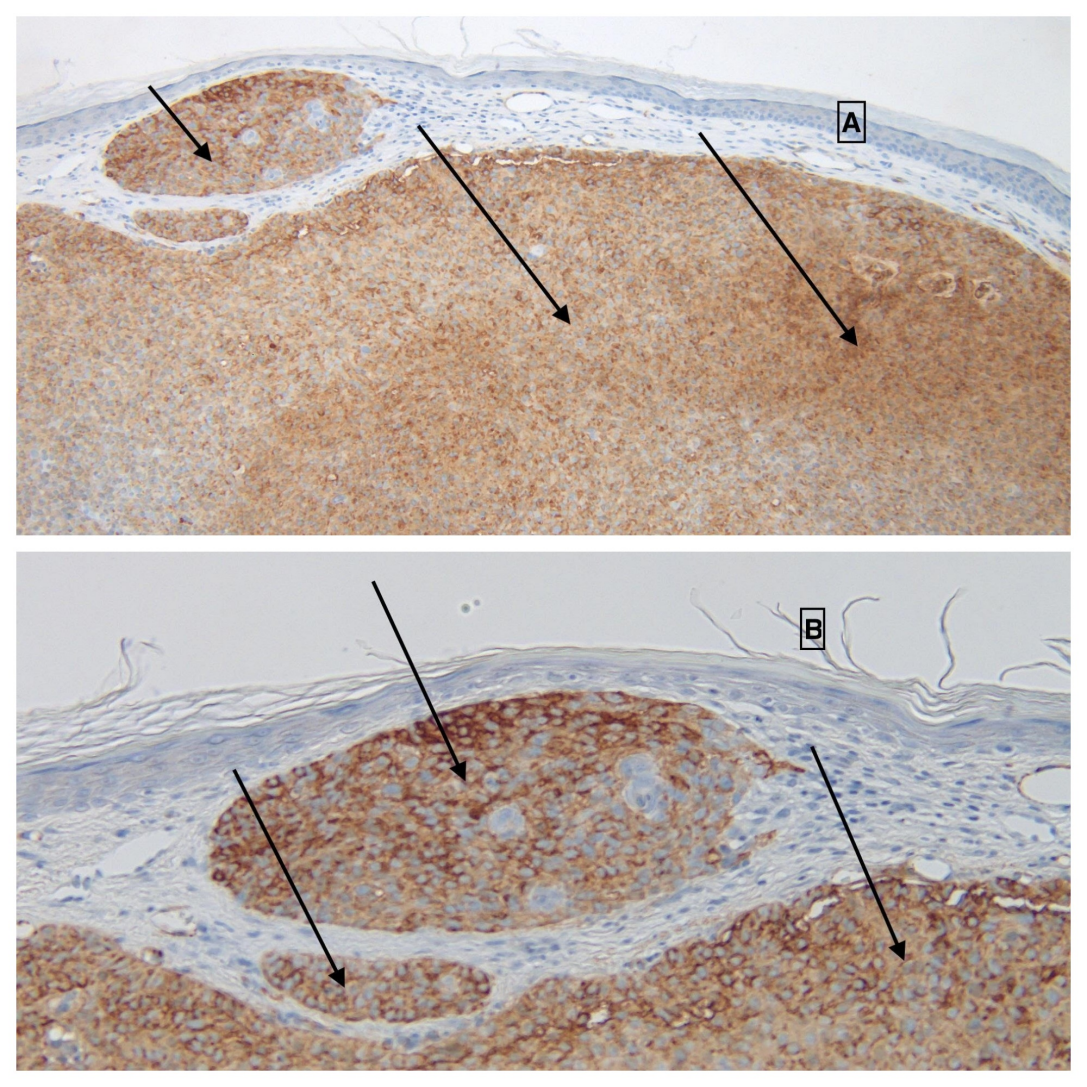
Immunoperoxidase studies of basal cell carcinoma with myoepithelial differentiation: smooth muscle actin. Distant (A) and closer (B) view of smooth muscle actin expression (solid arrows) of a basal cell carcinoma with myoepithelial differentiation. The diffuse positive actin staining of the tumor cells confirms their myoepithelial differentiation [Immunoperoxidase, smooth muscle actin: A, x2; B, x 10].

Immunoperoxidase studies were also performed. Ber-EP4 staining was focally positive within the tumor, confirming the diagnosis of basal cell carcinoma (Figure 3). Smooth muscle actin showed avid and diffuse positive staining of the tumor cells, thereby confirming myoepithelial differentiation (Figure 4). Sox10 immunostaining was negative, excluding the possibility of myoepithelioma.

Correlation of the clinical presentation, hematoxylin and eosin stained sections, and the immunoperoxidase studies established the diagnosis of basal cell carcinoma with myoepithelial differentiation. The tumor was excised in one stage using the Mohs micrographic surgery technique and the wound was treated with a side-to-side closure. There has been no recurrence at the one-year follow-up.

## Discussion

Basal cell carcinoma is the most common skin cancer. Unusual clinical variants (such as giant and red dot) and pathologic variants (such as clear cell, granular, and pleomorphic) of basal cell carcinoma have been described. In addition, divergent differentiation of basal cell carcinoma has been observed including tumors with eccrine, follicular, myoepithelial, neuroendocrine, sebaceous, and squamous features [1, 2, 4, 5].

Myoepithelial cells are specialized epithelial cells. They are present in the complex epithelium of several organs including the breast, the respiratory tract, and the salivary glands. They are located at the periphery of apocrine and eccrine sweat glands in the skin [2, 5, 10].

Seo et al are credited with the first description of basal cell carcinoma with myoepthelial differentiation in 1979; they reported a “basal cell carcinoma-signet ring type” on the nose of an 83-year-old man [7]. A decade later, in 1989, Sahin et al published the second patient with this tumor variant; they described a “basal cell carcinoma with hyaline inclusions” on the forehead of a 67-year-old woman [9]. Two years later, White et al reported this neoplasm as a “signet ring cell basal cell carcinoma” on the infraorbital area of a 63-year-old man [3]; that same year, in 1991, Suster and Ramon y Cajal published a case series of five individuals with “myoepithelial differentiation in basal cell carcinoma, emphasizing the accepted pathogenesis of this cancer [1]. In the subsequent 25 years, the features of only seven patients with basal cell carcinoma with myoepithelial differentiation have been published as individual case reports [2, 4-6, 8, 10].

Hence, basal cell carcinoma with myoepithelial differentiation is rare. Only five cases were accessed in the surgical pathology files of the Yale-New Haven Hospital from 1978 to 1990 and only one such neoplasm out of 139,846 basal cell carcinomas was discovered during a computerized search of the files of the Ackerman Academy of Dermatopathology from July 1, 1999 through June 30, 2010 [1, 2]. Including the patient in this report, basal cell carcinoma with myoepithelial differentiation has only been described in 16 patients (Table 1) [1-10].

**Table 1:** Clinical features of patients with basal cell carcinoma with myoepithelial differentiation. Abbreviations: C, case; CR, current report; mm, millimeters; Ref, reference; y, years. ^1^The tumor was located between the left angulus oculi medialis and the left lateral fibra nasi.

C	Age (y) Gender	Size (mm)	Appearance Morphology	Site	Differential diagnosis	Ref
1	43 Man	12x12	Not stated Nodule	Eyelid	Basal cell carcinoma	[1] C1
2	48 Man	3x3	Not stated Nodule	Cheek	Basal cell carcinoma	[2] C12
3	57 Man	5x5	Not stated Papule	Cheek	Squamous cell carcinoma	[1] C3
4	63 Man	10x7	Pearly Papule	Infraorbital	Basal cell carcinoma Trichoepithelioma	[3]
5	65 Man	10x10	Not stated Nodule	Eyelid	Basal cell carcinoma	[1] C2
6	67 Man	15x15	Not stated Mass	Cheek	Not stated	[4]
7	69 Man	4x4	Not stated Papule	Chin	Not stated	[2] C9
8	71 Man	3x3	Erythematous Papule	Forehead	Adnexal cyst Basal cell carcinoma Inflammatory papule	CR
9	74 Man	15x15	Skin-colored Plaque	Cheek	Not stated	[5]
10	79 Man	16x14	Pigmented Plaque	Upper lip	Not stated	[6]
11	83 Man	8x10	Pigmented Not stated	Nose	Not stated	[7]
12	45 Woman	4x4	Pearly Plaque	Upper lip	Not stated	[8]
13	49 Woman	8x8	Not stated Papule	Face	Angioma Nevus	[1] C4
14	52 Woman	7x7	Not stated Papule	Nose	Basal cell carcinoma	[1] C5
15	67 Woman	20x10	Ulcerated Not stated	Forehead	Not stated	[9]
16	69 Woman	35x28 x15	Ulcerated Nodule	Infraorbital^1^	Not stated	[10]

Basal cell carcinoma with myoepithelial differentiation has been reported in 11 men and five women. Hence, the tumor is a least twice as common in men as compared to women. At diagnosis, the patients ranged in age from 43 years to 83 years; the median age was 66 years.

The men ranged from 43 years to 83 years (median age was 67 years) at diagnosis; eight of the 11 men were older than age 60 when their tumor diagnosis was established. The women with basal cell carcinoma with myoepithelial differentiation were younger at diagnosis than the men; they ranged from 45 years to 69 years (median age was 52 years). Indeed, in contrast to the men, 60 percent of the women (three of five) were younger than 60 years of age when their tumor was diagnosed.

The onset of the basal cell carcinoma with myoepithelial differentiation in the reported patient was only two months; he had been seen for a complete skin examination three months earlier and there had not been a lesion present at that visit. However, similar to basal cell carcinomas without myoepithelial differentiation, these tumors are slow growing. The duration of tumor presence prior to evaluation and diagnosis has been as long as seven [10] to 10 [5] years.

The tumors ranged in size from 3 x 3 mm (Table 1, cases 2 and 8) to 3.5 x 2.8 x 1.5 cm (Table 1, case 16). Nearly two-thirds of the tumors (10 of 16) were smaller than 10 x 10 mm. The size of five of the carcinomas was between 1.0 to 2.0 cm.

The appearance of the carcinomas varied; two of the tumors were ulcerated. The color ranged from skin-colored (one patient) or pearly (two patients) to red (one patient) or pigmented (two patients). The appearance was not described in eight patients.

The most common tumor morphologies were papules (seven patients) and nodules (four patients). Less frequently, the carcinomas presented as a plaque (two patients) or mass (one patient). The morphology was not described in two patients.

Dermoscopic features of a basal cell carcinoma with myoepithelial differentiation were described for the gradually enlarging pigmented tumor that developed into a dome-shaped dark brown to black plaque on the right upper lip of a 79-year-old man. There were arborizing and irregular linear vessels. The vessels were intermingled with several dark brown areas in a whitish background [6].

All of the basal cell carcinomas with myoepithelial differentiation were located on the face. The cheek (four patients) and periocular area (four patients: eyelid for two patients and infraorbital for two patients) were the most common locations. Other frequent sites, each in two patients, included the forehead, nose, and upper lip. The remaining carcinomas were located on the chin and an unspecified site of the face.

The pathologic changes of basal cell carcinoma with myoepithelial differentiation show a multinodular dermal tumor that extends from the overlying epidermis and is characterized by two components. One of the components shows tumor nodules that demonstrate changes observed in a ‘typical’ or ‘conventional’ basal cell carcinoma. There are nodular aggregates of basaloid cells with palisading of the peripheral cells, focal areas of mucin within the tumor, and focal retraction of the surrounding stroma from the tumor nodules [1-10].

In addition, there are nodules of tumor cells that mimic a myoepithelioma. The tumor cells have eosinophilic cytoplasm. Many of the tumors have cells that are plasmacytoid or signet ring in appearance. The cells contain abundant cytoplasm or hyaline inclusions or both that causes the nucleus to be eccentric. Occasionally, as in the reported patient, these tumor aggregates surround mucin and have palisading of the cells at the periphery [1-10].

Basal cell carcinoma typically demonstrates positive immunofluorescence to cytokeratin, Ber-EP4, and p63. Myoepithelial cells found in neoplasms contain numerous intermediate filaments and microfilaments such as actin, cytokeratin, glial fibrillary acidic protein, myosin, and vimentin. The immunohistochemical staining patterns of basal cell carcinomas with myoepithelial differentiation express, to varying degrees, features of both basal cell carcinoma and myoepithelial cells (Table 2) [1-10].

**Table 2:** Basal cell carcinoma with myoepithelial features: immunohistochemical features. Abbreviations: Ber, Ber-EP4; C, case; Cal, calponin; CEA, carcinoembryonic antigen; CR, current report; Des, desmin; EMA, epithelial membrane antigen; GFAP, glial fibrillary acidic protein; Ker, keratin; NS, not stated; Ref, reference; SMA, smooth muscle actin (also include muscle specific actin); SMM, smooth muscle myosin; Sox, Sox10; Tot, total (positive staining/number tested); Vim, vimentin; +, positive staining; -, negative staining.

C a s e	B e r	C a l	C E A	D e s	E M A	G F A P	K e r	p 6 3	S 1 0 0	S M A	S M M	S o x	V i m	R e f
1	NS	NS	-	-	NS	+	+	NS	+	+	NS	NS	+	[1] C1
2	NS	NS	-	NS	-	NS	+	NS	-	+	NS	NS	-	[2] C12
3	NS	NS	-	-	NS	+	+	NS	+	+	NS	NS	+	[1] C3
4	NS	NS	NS	NS	NS	NS	+	NS	NS	-	-	NS	-	[3]
5	NS	NS	-	-	NS	+	+	NS	+	+	NS	NS	+	[1] C2
6	NS	NS	-	NS	+	+	+	NS	+	+	NS	NS	NS	[4]
7	NS	NS	-	NS	-	-	+	NS	-	-	NS	NS	-	[2] C9
8	+	NS	NS	NS	NS	NS	NS	NS	NS	+	NS	-	NS	CR
9	NS	+	NS	NS	+	NS	+	+	-	+	NS	NS	NS	[5]
10	+	+	-	-	-	-	+	+	+	+	NS	NS	+	[6]
11	NS	NS	NS	NS	NS	NS	+	NS	NS	NS	NS	NS	NS	[7]
12	NS	NS	NS	NS	NS	NS	+	NS	NS	+	NS	NS	-	[8]
13	NS	NS	-	-	NS	+	+	NS	+	+	NS	NS	+	[1] C4
14	NS	NS	-	-	NS	+	+	NS	+	+	NS	NS	+	[1] C5
15	NS	NS	NS	NS	NS	NS	+	NS	-	-	+	NS	+	[9]
16	NS	+	NS	NS	NS	NS	+	+	+	+	NS	NS	NS	[10]
Tot	2/2	3/3	0/9	0/6	2/5	6/8	15/15	3/3	8/12	13/15	1/2	0/1	7/11	

All of the basal cell carcinomas with myoepithelial differentiation showed positive staining for cytokeratin (15 of 15 tumors) and/or p63 (three of three tumors) and/or Ber-EP4 (two of two tumors). The tumors had positive expression for smooth muscle actin (or muscle-specific actin), glial fibrillary acid protein, S100, and vimentin: 87 percent (13 of 15 tumors), 75 percent (six of eight tumors), 67 percent (eight of 12 tumors), and 63 percent (seven of 11 tumors), respectively. Smooth muscle myosin (50 percent, one of two tumors) and epithelial membrane antigen (40 percent, two of five tumors) were less commonly expressed by the carcinomas. None of the basal cell carcinomas with myoepithelial differentiation demonstrated staining with carcinoembryonic antigen (zero of nine tumors), desmin (zero of six tumors), or Sox10 (zero of one tumor).

Electron microscopy was performed for three of the tumors [5, 7, 9]. The filaments that were observed provided a useful diagnostic feature to establish the myoepithelial differentiation of the tumor. Specifically, aggregates of filamentous material (haphazardly arranged at the center) created the globoid inclusion the displaced the cell’s nucleus. Neuroendocrine granules were absent [5].

The clinical differential diagnosis of basal cell carcinoma with myoepithelial differentiation (12 conditions) was only provided for eight patients. The most common diagnosis was basal cell carcinoma (six patients). Each of the following diagnoses was listed once: adnexal cyst, angioma, inflammatory papule, nevus, squamous cell carcinoma, and trichoepithelioma.

The pathologic differential of basal cell carcinomas with myoepithelial differentiation includes cutaneous benign mixed sweat gland tumor (also referred to as cutaneous pleomorphic adenoma), myoepithelioma, and myoepithelial carcinoma. Cutaneous mixed sweat gland tumor of the skin does not show the typical basal cell carcinoma component and has variable amounts of ductal structures within the chondromyxoid tumor stroma. Myoepithelioma is a benign dermal tumor composed only of myoepithelial cells without any basal cell carcinoma component and no epidermal connection that is usually Sox10 positive; in addition, myoepithelioma clinically occur in younger patients on the arms, in contrast to basal cell carcinoma with myoepithelial differentiation that occur in older individuals on the face. In contrast, myoepithelial carcinomas have similar pathologic features to myoepithelioma, but there is cytologic atypia mitoses [2, 4, 6, 10].

The pathologic differential diagnosis also includes other tumors that contain a prominent component of signet ring cells. These can include metastatic carcinomas (such as adenocarcinoma from the gastrointestinal tract and lobular carcinoma from the breast), melanoma, primary cutaneous plasmacytoid variant of squamous cell carcinoma and T-cell lymphoma. They also include cutaneous signet ring cell carcinoma of the eyelid [2, 10].

The pathogenesis of myoepithelial differentiation within a basal cell carcinoma remains to be established. Myoepithelial cells have the capacity for both epithelial and myxoid differentiation. Indeed, electron microscopy shows that the ultrastructure of a normal myoepithelial cell contains actin and myosin microfilaments, cytokeratin, and intermediate filaments. However, the tumor myoepithelial cells (which have also been referred to as ‘modified’ myoepithelial cells) possess a wide range of intermediate filaments and microfilaments; hence, the immunohistochemical reactivity of these cells can be variable, as demonstrated by pleomorphic expression of the basal cell carcinomas with myoepithelial differentiation [2, 10].

The treatment of basal cell carcinoma with myoepithelial differentiation was similar to that of conventional facial basal cell carcinomas of similar thickness without myoepithelial differentiation. The tumors were completely excised [4, 5, 10]. Indeed, similar to the reported patient, the cancer removal should preferably be performed using the Mohs micrographic surgical technique [2].

## Conclusions

Basal cell carcinoma with myoepithelial differentiation is a rare tumor. Including the patient described in this report, this variant of basal cell carcinoma has been described in 16 patients: 11 men and five women. The median age at diagnosis was 66 years; however, most of the men were older than 60 years whereas the majority of the women were younger than 60 years. Most of the tumors presented as small (less than 1.0 x 1.0 cm) papules or nodules. All of the carcinomas were located on the face. Their pathology demonstrated two components: one was that of a typical basal cell carcinoma (which demonstrates immunoreactivity for cytokeratin, Ber-EP4, and p63) and the other mimicked a myoepithelioma in which the tumor cells were plasmacytoid or signet ring in appearance and contained abundant eosinophilic cytoplasm or hyaline inclusions or both. The myoepithelial cells of the latter component contained numerous intermediate filaments and microfilaments; they had variable immunohistochemical expression that included not only cytokeratin but also actin, glial fibrillary acid protein, S100, and vimentin. The clinical impression of basal cell carcinoma with myoepithelial differentiation prior to biopsy was a basal cell carcinoma for 75 percent (six of eight) of the patients. The pathologic differential diagnosis of this tumor included cutaneous mixed sweat gland tumor of the skin, myoepithelioma, myoepithelial carcinoma, and tumors that contained a prominent signet ring cell component (such as metastatic gastrointestinal and breast carcinoma, melanoma, plasmacytoid squamous cell carcinoma, and T-cell lymphoma). Complete removal of the tumor, using the Mohs micrographic surgical technique, was recommended.
